# Mini‐review: Glucagon responses in type 1 diabetes – a matter of complexity

**DOI:** 10.14814/phy2.15009

**Published:** 2021-08-17

**Authors:** Mads Bisgaard Bengtsen, Niels Møller

**Affiliations:** ^1^ Department of Endocrinology and Internal Medicine Aarhus University Hospital Aarhus Denmark; ^2^ Department of Internal Medicine Regional Hospital Horsens Horsens Denmark

**Keywords:** alfa cells, Glucagon, hypoglycemia, type 1 diabetes

## Abstract

In recent years the role of altered alpha cell function and glucagon secretion in type 1 diabetes has attracted scientific attention. It is well established that glucagon responses to hypoglycemia are absent in type 1 diabetes, but more uncertain whether it is intact following other physiological and metabolic stimuli compared with nondiabetic individuals. The aim of this review is to (i) summarize current knowledge on glucagon responses during hypoglycemia in normal physiology and type 1 diabetes, and (ii) review human *in vivo* studies investigating glucagon responses after other stimuli in individuals with type 1 diabetes and nondiabetic individuals. Available data suggest that in type 1 diabetes the absence of glucagon secretion after hypoglycemia is irreversible. This is a scenario specific to hypoglycemia, since other stimuli, including administration of amino acids, insulin withdrawal, lipopolysaccharide exposure and exercise lead to substantial glucagon responses though attenuated compared to nondiabetic individuals in head‐to‐head studies. The derailed glucagon secretion is not confined to hypoglycemia as individuals with type 1 diabetes, as opposed to nondiabetic individuals display glucagon hypersecretion after meals, thereby potentially contributing to insulin resistance. The complexity of these phenomena may relate to activation of distinct regulatory pathways controlling glucagon secretion i.e., intra‐islet paracrine signaling, direct and autonomic nervous signaling.

## Graphical abstract

The absent glucagon responses to hypoglycemia in type 1 diabetes is a scenario specific to hypoglycemia, since other stimuli, including administration of amino acids, insulin withdrawal, lipopolysaccharide exposure, exercise and even meals lead to profound glucagon responses.

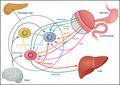

**What is already known?**

Glucagon responses to hypoglycemia are absent in type 1 diabetes.

**What this study has found?**

Here we have outlined that the absent glucagon responses to hypoglycemia in type 1 diabetes seems to be irreversible. This is a scenario specific to hypoglycemia, since other stimuli, including administration of amino acids, insulin withdrawal, lipopolysaccharide exposure, exercise and even meals lead to profound glucagon responses.

**What are the clinical implications of the study?**

The complexity of these phenomena may relate to activation of distinct regulatory pathways controlling glucagon secretion i.e., intra‐islet paracrine signaling, direct and autonomic nervous signaling. In our eyes, this paper highlights an important issue in type 1 diabetes – a derailed glucagon secretion, and hopefully inspires to further research into the glucagon conundrum of type 1 diabetes.

## 1 INTRODUCTION

Much research has focused on insulin and the autoimmune destruction of the beta cells involved in type 1 diabetes. However, especially within recent years the role of altered alpha cell function and glucagon secretion in type 1 diabetes has attracted considerable interest (Yosten, 2018). Lack of insulin and excess of glucagon are believed to be main triggers of the metabolic disarray in type 1 diabetes, in particular derailed endogenous glucose production, ketogenesis, and ureagenesis, as proposed by RH Unger et al in the *bihormonal hypothesis* (Unger, 1985). In general, these processes are self‐perpetuating and poor metabolic control and insulin deficiency increase circulating glucagon concentrations, a scenario which can inflict diabetic ketoacidosis (Svart et al., 2016; Voss et al., 2018). Glucagon is recognized as a counter regulatory hormone released during stress conditions, such as hypoglycemia, exercise, systemic inflammation and fasting (Finan et al., 2020; Walker et al., 2011). It is well established that glucagon responses to hypoglycemia are severely impaired in type 1 diabetes (Bengtsen et al., 2020; Cryer et al., 2001; Gerich et al., 1973) but whether glucagon secretion is completely intact following other physiological and metabolic stressful stimuli compared with nondiabetic individuals has been less thoroughly investigated. The aim of this mini‐review was to: (i) summarize the current knowledge on glucagon responses during hypoglycemia in normal physiology and type 1 diabetes, and (ii) review human *in vivo* studies investigating glucagon responses during various stressful conditions in individuals with type 1 diabetes compared with the physiological response in nondiabetic individuals. The current review is prompted by a series of human trial conducted at our research facility (Bengtsen et al., 2020; Rittig et al., 2015; Svart et al., 2016; Voss et al., 2018), and all data have previously been published.

## 2 REGULATION AND ACTIONS OF ALPHA CELL GLUCAGON

**FIGURE 1:**
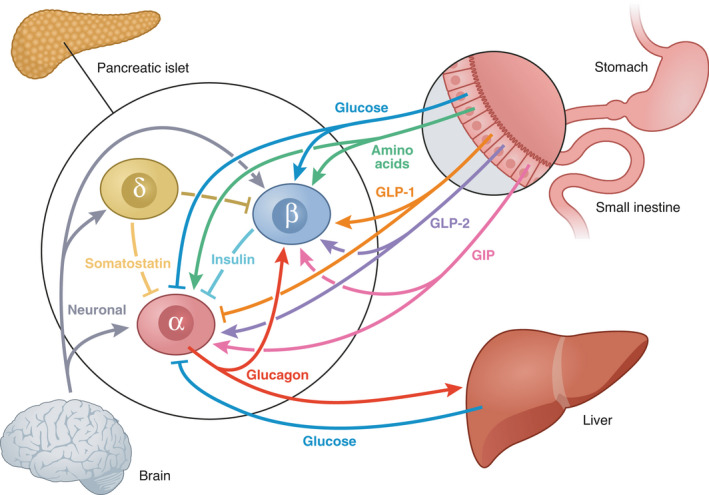
The alpha cell glucagon regulation. Alpha cell glucagon secretion is regulated by nutrients (amino acids), indirectly (and perhaps directly) by glucose, the autonomic nervous system (neuronal innervation from the brain), gut incretins and direct/indirect paracrine signaling from beta cells and delta cells within the pancreatic Islets of Langerhans. α = alpha cell. Β = beta cell. Δ = delta cell. GLP‐1, Glucagon‐like peptide 1; GLP‐2, Glucagonotropic glucagon‐like peptide 2; GIP, Glucose‐dependent insulinotropic polypeptide

Multiple mechanisms are involved in the complex regulation of alpha cell glucagon secretion including nutrients, the autonomic nervous system, gut incretins and direct/indirect paracrine signaling from beta cells and delta cells in the pancreatic islets of Langerhans (Cryer, 2012; Yosten, 2018) (Figure 1). The microstructure within the islets includes endocrine cell types intermingled with each other with beta cells being the most prevalent. Early studies have shown that the intraislet blood microcirculation flows from the beta cell to the alpha cell (Stagner & Samols, 1992) supporting the traditional concept that alpha cell glucagon secretion is critically dependent on upstream signals from beta cells both during hypoglycemia and hyperglycemia (Yosten, 2018). However, the concept of microcirculation is debated and other mechanisms clearly contribute. The intraislet insulin hypothesis, which has attracted considerable research interest, proposes that a decrease or increase in insulin secretion from the beta cell is a reciprocal signal in itself to alter the tonic intraislet insulin inhibition of alpha cells leading to either increased or decreased glucagon secretion (Cooperberg & Cryer, 2010). While the intra islet insulin hypothesis might be an important component of iatrogenic hypoglycemia other regulatory mechanism within the islets exist including somatostatin from the delta cells inhibiting both glucagon and insulin secretion in a paracrine fashion. Other prominent regulatory pathways include the gut‐pancreas axis and incretin hormones. This regulatory pathway consist of glucose‐dependent insulinotropic polypeptide (GIP) from intestinal K cells and glucagonotropic glucagon‐like peptide 2 (GLP‐2) from intestinal L cells both inducing glucagon secretion whereas glucagon‐like peptide 1 (GLP‐1) from intestinal L cells inhibits glucagon secretion (Knop, 2018). GIP receptors are expressed in alpha cells, whereas GLP‐1 receptors are not suggesting direct effects of GIP and indirect effects of GLP‐1 on glucagon release (Knop, 2018). Interestingly, a recent paper reported that the glucagonostatic responses to both glucose and GLP‐1 are impaired in individuals with 1 diabetes (Bagger et al., 2021). Furthermore, as outlined in a recent review (Rickels, 2019) and supported by human studies of nondiabetic individuals (Mundinger et al., 2016) activation of the autonomic nervous system governed by central and peripheral glucose‐sensing neurons also plays a role in the regulation of glucagon secretion. Finally, mixed‐nutrient meals increase blood glucose, which indirectly, e.g., through insulin, or perhaps directly inhibit glucagon secretion: Rodents studies have led to the theory of alpha cells possessing an intrinsic glucose sensing ability via the glucose sensor protein glucokinase (Heimberg et al., 1996). Amino acids (especially alanine and arginine) are well‐known direct stimulators of both the alpha cell and beta cell (Felig et al., 1969). Recent work have highlighted the existence of a liver alfa‐cell axis and reported that this axis is compromised in individuals with type 2 diabetes and prediabetic states in terms of failure of the liver to increase ureagenesis in response to glucagon (Albrechtsen et al., 2019). Furthermore, in parallel with beta cells, alpha cells harbor several key signaling components, i.e., GLUT1, glucokinase, and the KATP channel; however, compared to beta cells, alpha cells exhibit a different expression and localization of many ion channels, such as voltage‐dependent Na^+^ channels and T‐, L‐, and P/Q‐type Ca^2+^ channels, generating a substantially different electrophysiologic profile (Walker et al., 2021). Overall, the regulation is complex and involves several potential feedback loops as depicted Figure 1.

The actions of glucagon are best documented during hypoglycemia where glucagon is characterized by its ability to increase endogenous glucose production by increasing gluconeogenesis and glycogenolysis, hence ensuring sufficient supply of glucose to vital tissues, particularly the central nervous system and muscles (Sandoval & D’Alessio, 2015). As proposed in a recent review (Finan et al., 2020) glucagon secretion also plays an essential role during physiological and metabolic stress where increased energy supply is warranted (Finan et al., 2020). Cortisol, epinephrine and norepinephrine from the adrenal glands, and growth hormone from the pituitary gland are secreted and act in parallel with development of symptoms of hypoglycemia (Cryer et al., 2001). The main target organ for glucagon is the liver which is reached via the hepatic portal vein system. Here, glucagon exerts its effect on the G‐protein‐coupled receptors stimulating glycogenolysis and gluconeogenesis (Felig et al., 1969; Finan et al., 2020; Sandoval & D’Alessio, 2015). Glucagon also stimulates hepatic fat oxidation, ketogenesis, protein catabolism, and ureagenesis (Holst et al., 2017). The counter regulatory metabolic response is highly efficient in people without diabetes, whereas individuals with type 1 diabetes are more susceptible to hypoglycemia due to several factors, including chronic sustained hyperinsulinemia (Gregory et al., 2019), repeated episodes of hypoglycemia impairing the hormonal counter regulatory response and attenuating symptoms to subsequent hypoglycemia (i.e., hypoglycemia‐associated autonomic failure) and due to severely impaired glucagon responses to hypoglycemia as outlined below.

## 3 IMPAIRED GLUCAGON RESPONSES TO HYPOGLYCEMIA

Originally reported by Gerich et al in 1973 (Gerich et al., 1973), the lack of any glucagon counter regulatory response to hypoglycemia has consistently been confirmed in individuals with type 1 diabetes (Bengtsen et al., 2020; Cryer et al., 2001). In the past, long diabetes duration was considered a prerequisite for the lack of glucagon response to occur but mounting evidence suggest that it often occurs as early as the first months to years after the diagnosis of type 1 diabetes (Siafarikas et al., 2012). Although often associated with hypoglycemia‐associated autonomic failure (HAAF), the absence of glucagon response to hypoglycemia is present in some people with type 1 diabetes with otherwise intact hormonal counter regulatory responses (Cryer, 2004). The loss of glucagon responses to hypoglycemia seems irreparable in the course of the disease, and despite scrupulously avoidance of hypoglycemia for four months the glucagon response remains absent whereas epinephrine and hypoglycemic symptoms rematerialize (Cranston et al., 1994), supporting the notion that the loss of glucagon responses to hypoglycemia is irreversible. Only whole pancreas transplantation seems capable of restoring glucagon responses to hypoglycemia (Diem et al., 1990), while islet transplantation have shown discordant results with potential partial restoration of glucagon responses to hypoglycemia (Rickels et al., 2005). There is also evidence that the glucagon response is lost in advanced type 2 diabetes with severe endogenous insulin deficiency (Segel et al., 2002). It therefore appears conceivable that alpha cell dysfunction is closely linked to the development of beta cell failure in diabetes. However, one study (Sherr et al., 2013) failed to establish a significant association between residual beta cell function and glucagon responses to hypoglycemia in young people with type 1 diabetes whereas some have found an association between decreasing glucagon responses and diabetes duration (Siafarikas et al., 2012).

## 4 POTENTIAL MECHANISMS BEHIND THE LOSS OF GLUCAGON RESPONSE TO HYPOGLYCEMIA

Alpha cell insensitivity or irresponsiveness to paracrine signaling from the beta cell have been suggested to be involved in the lack of glucagon secretion in response to hypoglycemia (Banarer et al., 2002). This hypothesis does not exclude the possibility of other intra islet mechanisms and studies in rodents have led to the hypothesis that normal pancreatic alpha cells possess intrinsic glucose sensing abilities and that these are redundant in type 1 diabetes (Le Marchand & Piston, 2010). Additionally, it has been suggested that excessive delta cell somatostatin secretion in the intra islet milieu augments suppression of alpha cells leading to absent glucagon responses during hypoglycemia (Karimian et al., 2018). These theories, based on rodent studies, remain to be confirmed in humans. Islet autonomic innervation may also be involved in the regulation of glucagon secretion, although it does not appear to be critical for glucagon secretion as humans with spinal cord transections (Palmer et al., 1976) and the denervated transplanted human pancreas (Diem et al., 1990) secrete glucagon when subjected to hypoglycemia. In conclusion, these studies indicate that the mechanisms behind the absent glucagon secretion in type 1 diabetes appear to reside within the islets of Langerhans.

## 5 GLUCAGON RESPONSES TO INSULIN WITHDRAWAL AND FASTING

**FIGURE 2:**
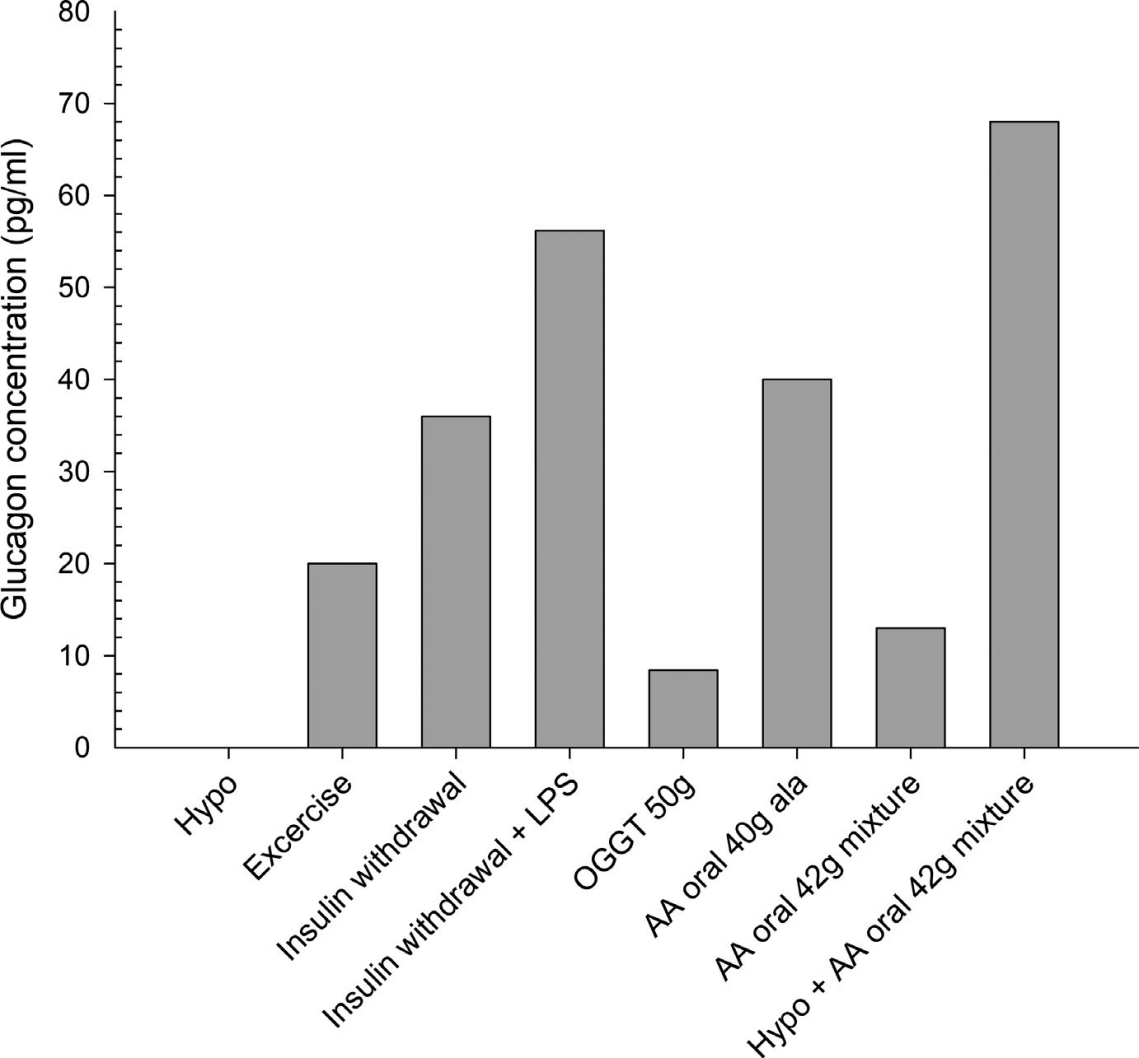
Glucagon response (pg/ml) to specific stimuli in type 1 diabetes. Hypoglycemia (Hypo) (Bengtsen et al., 2020; Gerich et al., 1973) does not elicit a glucagon responses in individuals with type 1 diabetes whereas exercise (Mallad et al., 2015), insulin withdrawal (Moller et al., 2006; Voss et al., 2018) and insulin withdrawal combined with lipopolysaccharide administration (insulin withdrawal +LPS) (Svart et al., 2016) lead to profound glucagon responses. The paradoxical glucagon increase following a meal is evidenced by an oral glucose tolerance test with 50 gram glucose (OGGT 50g) (Hare et al., 2010) and by orally administered 40 gram alanine (AA oral 40g ala) (Wiethop & Cryer, 1993) or 42 gram mixture of amino acids (AA oral 42g mixture) (Rosetti et al., 2008). Oral ingested amino acids during hypoglycemia lead to a recovery of glucagon responses in individuals with type 1 diabetes (Hypo + AA oral 42g mixture) (Rosetti et al., 2008). Glucagon concentration was calculated by average peak concentration minus average baseline concentration. Due to varying study designs the exact increase in glucagon cannot be accurately compared

In humans, glucagon concentrations are substantially increased following insulin withdrawal/a state of insulinopenia (Moller et al., 2006). As previously reported (Voss et al., 2018) individuals with type 1 diabetes continuously secrete plasma glucagon after 14 hours of insulin withdrawal despite mean plasma‐glucose of around 20 mmol/L (Figure 2); and following intravenous insulin infusion glucagon concentrations are suppressed. These findings support the theory of intraislet alpha cell and beta cell interaction implying that when insulin is absent there is no negative feedback on glucagon secretion and when insulin is present alpha cell glucagon secretion is inhibited (Cooperberg & Cryer, 2010). Supporting this concept fasting concentration of glucagon have been found in some but not in all studies to be elevated in individuals with type 1 diabetes compared with nondiabetic individuals (Raskin & Unger, 1978). Assuming a state of relative nocturnal intraislet hypoinsulinaemia this could explain the ensuing morning hyperglucagonemia.

## 6 GLUCAGON RESPONSES TO AMINO ACID EXPOSURE

It has previously been reported that glucagon responses to specific oral amino acid such as alanine (Wiethop & Cryer, 1993), a mixture (Rosetti et al., 2008) or intravenous administered alanine (Porcellati et al., 2007) are preserved in type 1 diabetes. Administration of amino acids lead to varying glucagon responses depending on several factors including dosage, administration route and serum insulin concentration (Figure 2). It is evident that glucagon concentrations may rise above those observed during hypoglycemia in nondiabetic individuals indicating that alpha cells are highly responsive to amino acids. Importantly, administration of amino acids during hypoglycemia stimulates glucagon secretion in individuals with type 1 diabetes despite substantial hyperinsulinemia (Porcellati et al., 2007; Rosetti et al., 2008) reflecting the presence of intact alpha cell function in response to amino acids. As shown in Figure 2, hypoglycemia potentiates the stimulatory effect of amino acids on the alpha cells (Rosetti et al., 2008) indicating a synergistic effect. The reason is unclear but amino acids affect both beta and alpha cells potentially leading to alterations in the paracrine cell‐to‐cell signaling of course taking into account the fewer amount of beta cells in individuals with type 1 diabetes. Furthermore, oral amino acids also affect the incretin hormones in the gut (Dunning et al., 2005) thereby possibly affecting alpha and beta cells in an indirect manner.

## 7 GLUCAGON RESPONSES TO INFLAMMATION AND EXERCISE

It has been known for several years that systemic infection and inflammation is associated with high circulating concentrations of glucagon (Rocha et al., 1973) and that this is related to cytokines such as TNF‐α (Van der Poll et al., 1991). As previously reported (Svart et al., 2016), lipopolysaccharide infusion combined with insulin reduction resulted in profound glucagon responses in individuals with type 1 diabetes (Figure 1), resembling those seen in nondiabetic individuals (Rittig et al., 2015), and demonstrating intact glucagon responsiveness to systemic inflammation. It is uncertain how lipopolysaccharide exposure creating an acute systemic inflammation stimulates glucagon secretion. Potentially cytokine surges play a role and some studies have documented catecholamine receptors on alpha cells supporting the theory that glucagon secretion could be activated either through a systemic catecholamine response or by direct neuronal islet innervation (Schuit & Pipeleers, 1986). The normal physiological response to exercise is characterized by a decrease in blood glucose followed by a compensatory increased glucagon secretion and increased hepatic glucose production (Burton et al., 2004). This glucagon response appears to be preserved yet attenuated in individuals with type 1 diabetes compared with nondiabetic individuals (Mallad et al., 2015), implying that glucagon release during exercise is activated by other regulatory pathways than those prevailing during hypoglycemia. Moreover, in individuals with type 1 diabetes exercise leads to a paradoxical increase in insulin concentrations believed to be caused by increased blood flow and absorption of subcutaneous deposits of insulin possibly contributing to the attenuated glucagon release (Mallad et al., 2015).

## 8 GLUCAGON RESPONSES TO MIXED‐MEAL TESTS AND ORAL GLUCOSE TOLERANCE TEST

It has been reported that individuals with type 1 diabetes, as opposed to nondiabetic individuals, secrete glucagon after mixed‐meal stimulations (Sheer et al., 2014) and oral glucose tolerance tests (Hare et al., 2010). These apparently paradoxical findings plausibly relate to an absent increase in beta cell insulin secretion following a meal and subsequent failure to convey a decrease in alpha cell glucagon secretion. One study (Sheer et al., 2014) reported that individuals with type 1 diabetes had normal glucagon suppression after an intravenous compared with an oral glucose test, leading to the authors suggesting defective incretin mediated regulation of glucagon secretion in type 1 diabetes. The excessive glucagon secretion following a meal could relate to low paracrine pancreatic insulin levels and supports the hypothesis that hyperglucagonemia plays a significant role in the pathogenesis of hyperglycemia leading to tissue glucotoxicity and thereby insulin resistance in type 1 diabetes (Unger & Cherrington, 2012).

## 9 GLUCAGON MAXIMUM STIMULATORY CAPACITY

In spite of preserved glucagon secretion after specific stimuli in adults with type 1 diabetes, the maximum glucagon secretory capacity of these individuals in head‐to‐head studies is reduced compared with matched individuals without diabetes (Porcellati et al., 2007; Rosetti et al., 2008; Wiethop & Cryer, 1993). The reason for the attenuated glucagon response at physiological blood glucose levels with similar peripheral insulin concentrations remains unclear. One possibility is that the chronic hyperinsulinemia present in type 1 diabetes is involved (Gregory et al., 2019) but other mechanisms may certainly also contribute.

## 10 GLUCAGON FROM EXTRAPANCREATIC TISSUES

Finally, it has generally been assumed that glucagon is exclusively secreted from the pancreatic alpha cells. Several studies however have measured glucagon in pancreatectomized animals and humans with discordant results (Unger et al., 1966), and recently it was shown that pancreatectomized patients display clear‐cut glucagon increments following an oral glucose tolerance test with peak concentrations appropriate for hormonal counter regulation (Lund et al., 2016). The authors speculate that enteroendocrine cells in the gastrointestinal tract may secrete glucagon. If this holds true and there is no analytical cross‐reactivity, it is evidently possible that the glucagon responses referred to above may to some extent derive from the gastrointestinal tract. The physiological role of glucagon from extrapancreatic tissues is yet to be determined.

## 11 CONCLUSION

In summary, available data suggest that in people with type 1 diabetes the loss of glucagon secretion after hypoglycemia is irreversible. This scenario seems to be specific to hypoglycemia, since application of other stimuli, including administration of intravenous and oral amino acids, insulin withdrawal, lipopolysaccharide exposure and exercise lead to profound glucagon responses though attenuated compared to nondiabetic individuals in head‐to‐head studies. The derailed glucagon secretion is not only confined to hypoglycemia as individuals with type 1 diabetes, as opposed to nondiabetic individuals, display glucagon hypersecretion after meal tests, thereby possibly contributing to hyperglycemia and insulin resistance. The complexity of these phenomena may relate to activation of distinct regulatory pathways controlling glucagon secretion i.e., intraislet paracrine signaling, direct signaling and autonomic signaling and certainly merits further investigations into the unsolved conundrum of glucagon secretion in type 1 diabetes.

## CONFLICTS OF INTEREST

The authors declare that there is no duality of interest associated with this manuscript.

## AUTHOR CONTRIBUTION

MBB wrote the first draft of the manuscript; MBB and NM designed the figures, and both contributed to the final version of the manuscript.
